# Sporadic medullary thyroid cancer: a systematic review and meta-analysis of clinico-pathological and mutational characteristics predicting recurrence

**DOI:** 10.1186/s13044-022-00130-8

**Published:** 2022-07-22

**Authors:** Benjamin Cosway, Jonathan Fussey, Dae Kim, James Wykes, Michael Elliott, Joel Smith

**Affiliations:** 1 Newcastle Hospitals NHS Foundation Trust, Newcastle, UK; 2 University Hospital of Birmingham, Birmingham, UK; 3 Royal Marsden Hospital, London, UK; 4 Chris O’Brien Lifehouse, Sydney, Australia; 5 Royal Devon and Exeter Hospital, Exeter University, Exeter, UK

**Keywords:** Sporadic medullary thyroid cancer, Recurrence, Systematic review

## Abstract

**Introduction:**

Sporadic medullary thyroid cancer accounts for 75% of all medullary thyroid cancers and presents at a more advanced disease stage than its hereditary counterparts. Yet there is little evidence to support risk stratification of patients according to risk of recurrence.

**Methods:**

A systematic review and meta-analysis was performed investigating clinical and pathological factors that are associated with recurrent disease in patients with medullary thyroid cancer.

**Results:**

10 studies totalling 458 patients were included in the meta-analyses. T3 and T4 disease (OR 9.33 (95% CI 2.5 – 34.82) p = 0.0009.), AJCC stage III and IV disease (OR 13.34 (95% CI 2.9 – 60.3) p = 0.0008) and the presence of nodal disease (OR 7.28 (95% CI 7.2–43.3) p = 0.03) were all associated with recurrent disease. *RET mutations* (OR 0.08 (95% CI -0.03–0.19) p = 0.17) *and RET* 918 T mutations (OR 1.77 (95% CI 0.804.0) P = 0.17) were not associated with disease recurrence. It was not possible to pool data with respect to extrathyroidal extension, extracapsular extension, peri-neural and lymphovascular invasion and *RAS* mutations.

**Conclusion:**

T3 and T4 disease, AJCC stage III and IV disease and the presence of nodal disease are associated with recurrent disease. The heterogeneous reporting of recurrence and the lack of individual patient data precludes larger scale meta-analyses. Future research in this area should involve collaboration to establish standardised definitions of disease recurrence.

**Supplementary Information:**

The online version contains supplementary material available at 10.1186/s13044-022-00130-8.

## Introduction

### Rationale

Medullary thyroid cancer (MTC) is a neuroendocrine tumour of the parafollicular C cells accounting for 1–2% of thyroid cancers [1]. Whilst 25% of MTC is hereditary and caused by germline variants in the *RET* proto-oncogene, the majority occurs sporadically (sMTC) [2]. Unlike hereditary MTC where prophylactic surgery can be offered based on germline *RET* mutation status, sMTC presents clinically or incidentally, often with advanced disease with regional and distant disease rates at presentation of 35% and 13% respectively [3]. As a result, sMTC has a poorer overall survival at 10 years in comparison with hereditary disease [4].

Whilst there is broad consensus on the management of node negative sMTC with a total thyroidectomy and central nodal clearance, there are international divergences. For example, there are subtle differences in the role of prophylactic neck dissection as well as the role of hemithyroidectomy alone for the management of small tumours [1, 5]. This highlights the some of the uncertainty in identifying patients who may be at higher risk of recurrent disease.

Broadly speaking, patients with sMTC have one of three potential responses to initial treatment which can impact on prognosis; excellent, biochemically incomplete or structurally incomplete response [6]. Whilst the presence of a recordable and rising calcitonin, as well as persistent structural disease impacts on prognosis, a better understanding of why some sMTCs recur following treatment despite an excellent response to initial therapy may allow for high risk patients to be targeted for more aggressive treatment on an individual basis. Whilst a systematic review and meta-analysis has investigated factors predicting recurrent disease in sMTC, this study was contaminated by the pooling of patients with recurrent, persistent and progressive disease [7]. Therefore, the extent to which we can make these decisions based on the published data is not clear. This review aims to provide an analysis of factors that may aid in identifying patients at risk of recurrence and therefore who may benefit from more intensive therapeutic strategies.

### Objectives

We aim to investigate the relevance of clinicopathological factors that influence recurrence in patients with sMTC. The PICO criteria for this study are outlined below:

### Participants

Participants were patients with a diagnosis of sporadic medullary thyroid cancer.

### Intervention

There was no specific intervention being measured. However, we measured a number of clinicopathological factors including TNM staging, disease stage, extrathyroidal extension, extracapsular extension, peri-neural and lymphovascular invasion and *RET/RAS* mutation status. We did not expect all studies to report on all characteristics.

### Comparator

Comparator groups were patients without the exposure of interest that may influence recurrence.

### Outcomes

The primary outcome was the presence of recurrent disease at any time point. There is no standardised definition of recurrence for sMTC. Recurrence in this study was defined as clinical or radiological evidence of sMTC during clinical follow up following an ‘excellent response’ to treatment [6]. Therefore there had been no clinical, biochemical or radiological evidence of persistent disease within 6 months following primary surgery. This is consistent with definitions used by other authors [8].

## Methods

### Protocol and registration

This review is registered with the PROSPERO international prospective register of systematic reviews; CRD42020179396.

### Eligibility criteria

Studies were eligible for inclusion based on the PICO criteria outlined in the objectives. Studies were excluded if they were not English language, not published in the last 25 years, commentaries/editorials/reviews, case studies or series of < 10 patients with sMTC or where data on sMTCs could not be extracted. Where data on patients from a single institution were published multiple times, the most complete dataset for pathological characteristics was included.

### Information sources

The Cochrane Library, Medline (including PubMed indexed citations) and EMBASE databases were searched until 09/05/21. Bibliographic searches of studies identified for inclusion were also undertaken until 09/05/2021.

### Search

Search criteria for the MEDLINE database is shown in additional file 1.

### Study selection

Searches were performed by BC. Subsequent screening and review of full test articles were were performed by both BC and JF. Any discrepancies were resolved by discussion including the senior author JS.

### Data collection processes

Data extraction was performed using a purpose designed and piloted proforma written by BC. Data extraction was performed independently by BC and JF with any discrepancies resolved by discussion including the senior author. JS Where data extraction was not possible, we made contact once by email to the corresponding author.

### Data items

Data extraction included the author, year of publication, average age, sex and number of patients. The clinico-pathological data extracted included pathological Tumour-Node-Metastasis (TNM) stage, AJCC stage (both extracted as or converted to the 7^th^ edition), somatic *RET* and *RAS* mutation status, presence of extranodal extension, extrathyroidal extension, lymphovascular and perineural invasion. The primary outcome was the presence of clinical or radiological evidence of recurrent disease following an excellent response to treatment.

Persistent disease was not considered as recurrent disease as it has been in other meta-analyses [7].

### Risk of bias in individual studies

There is no internationally recognised gold standard for bias assessment of non-interventional observational studies. The Newcastle–Ottawa scale has been used in a previous review looking at the impact of molecular genetics on clinical management of sporadic medullary thyroid cancer [9]. This was therefore chosen for bias assessment in this systematic review. These assessments were performed by BC and JF.

### Summary measures

The Review manager 5.3 software (Cochrane Collaboration, UK) was used for statistical analysis. Pooled estimates of odds ratios and their 95% confidence intervals were calculated using a random effects model to help account for within study heterogeneity.

### Synthesis of results

Heterogeneity was assessed using the chi-squared test, I^2^ statistic and visual inspection of the forest plots. A significance level of < 0.1 was interpreted as evidence of heterogeneity. Where levels of inconsistency were above 50%, a ‘one out’ sensitivity analysis was performed to test the robustness of the overall results. This was performed with respect to expected and observed study heterogeneity. In our scoping of the literature, we anticipated heterogeneity in the form of.

1. Differing lengths of follow up
2. Different surgical and non-surgical management strategies over the 25 years of included literature
3. Study quality (risk of bias)
4. Sample sizes

### Risk of bias across studies

Publication bias was planned to be assessed using funnel plots where appropriate.

## Results

### Study selection

We identified 235 individual studies from our search strategy and after initial screening identified 99 papers for full text review. Contact was made by email to 31 groups who had published a total of 39 papers for further data. This was provided by one group and was subsequently included in our pooled analyses [4]. We were unable to establish contact with the other authors. Therefore, there were a total of 56 manuscripts, many of which were large series, where data on recurrent disease could not be extracted with respect to individual clinico-pathological patient characteristics. In total, 10 studies with 458 patients were included in our meta-analyses.

### Study characteristics and risk of bias

Ten studies were identified for pooled analyses in this systematic review. The characteristics, as well as their Newcastle Ottawa bias assessment scores, are found in Table 1.

**Table 1:** Characteristics and risk of bias in included studies

1^st^ Author and Year		Number of Patients (number of recurrences)	Males	Average Age	Patient Selection				Data Collection			Total Score
					Adequate Case Definition?	Consecutive cases?	Appropriate Comparators?	Clear comparator definition?	Record linkage?	Length of follow up Average > 2 years?	< 10% loss to follow up?	
Saltiki [4]	2019	163 (86)	73	Mean 52.7	✓	﻿✓	﻿✓	﻿✓	﻿✓	Mean 7.5 years (for sMTC and familial)	﻿✓	7
Grubbs [10]	2016	62 (16)	36	Mean 50.1	﻿✓	?	﻿✓	﻿✓	﻿✓	Median 10.5 years	﻿✓	6
Simbolo [11]	2014	20 (9)	9	Mean 51.5	﻿✓	?	﻿✓	﻿✓﻿	﻿✓	Median 4 years	﻿✓	6
Abraham [8]	2011	26 (3)	14	Mean 55.1	﻿✓	?	﻿✓	﻿✓	﻿✓	Mean 6.25 years	﻿✓	6
Dvorakova [12]	2008	48 (31)	19	Mean 54.1	﻿✓	?	﻿✓	﻿✓	﻿✓	Mean 2.5 years	﻿✓	6
Raffell [13]	2004	15 (1)	3	51	﻿✓	?	﻿✓	﻿✓	﻿✓﻿	Mean 4.6 years	﻿✓	6
Schilling [14]	2001	34 (23)	44	Mean 58.9	﻿✓	?	﻿✓	﻿✓	﻿✓﻿	Mean 6.4 years	﻿✓	6
Uchino [15]^1^	1999	38 (4)	?	?	﻿✓	?	﻿✓	﻿✓﻿	﻿✓	Median 11 years	﻿✓	6
Uchino [16] ^2^	1998	34 (4)	?	?	﻿✓	?	﻿✓	﻿✓	﻿✓	Median 11 years	﻿✓	6
Romei [17]	1996	18 (4)	4	Mean 58.9	﻿✓	?	﻿✓	﻿✓	﻿✓	Mean 2.7 years	﻿✓	6

1. Used for overall RET status alone. 2. Used for M198T status alone. This was due to how the data was reported. There was no crossover of patients

**Fig. 1:**
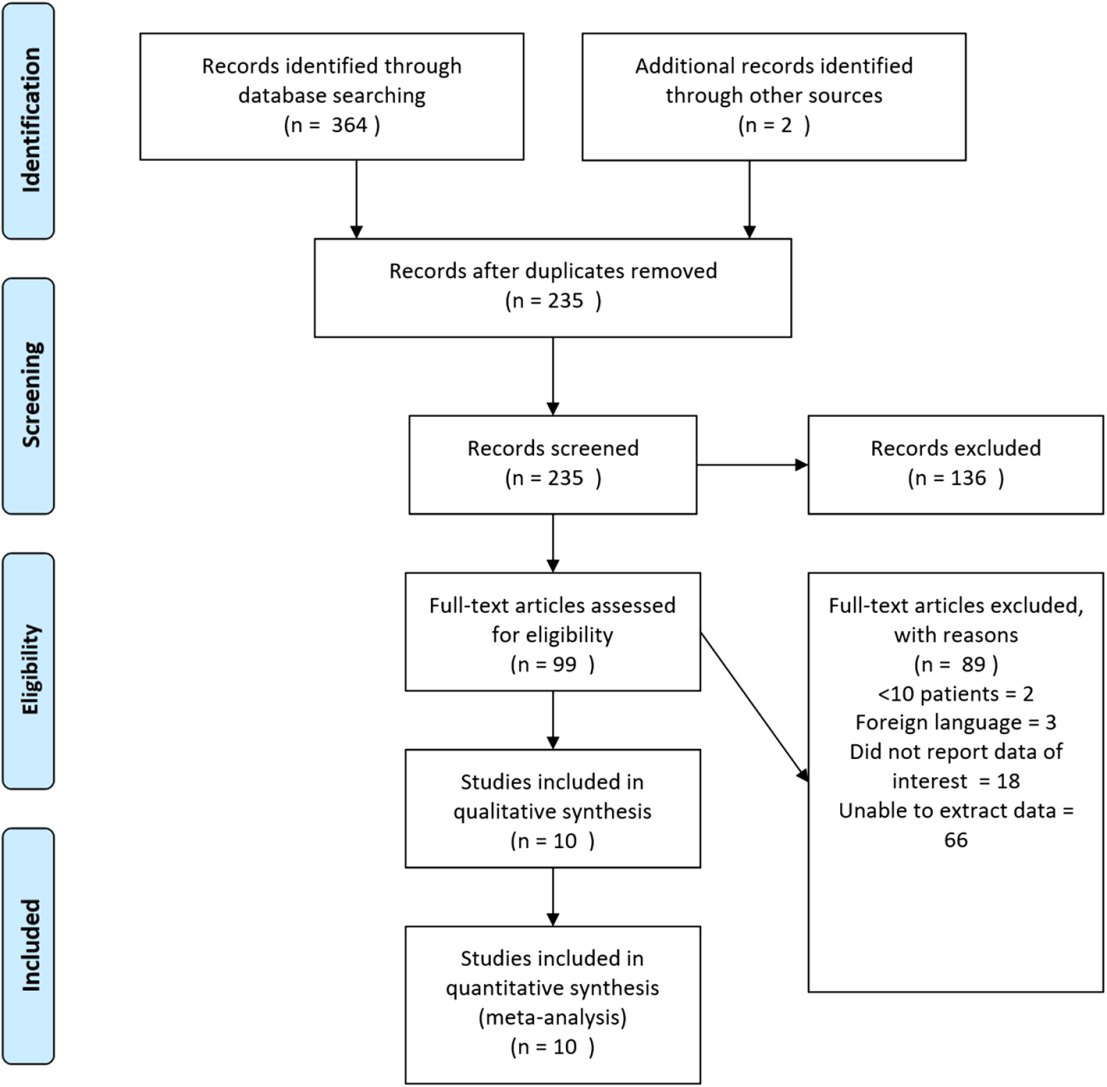
Prisma flow chart

Our PRISMA flow chart outlining our study selection is shown in Fig. 1.

### Results of individual studies and synthesis of results

#### T stage

**Fig. 2:**
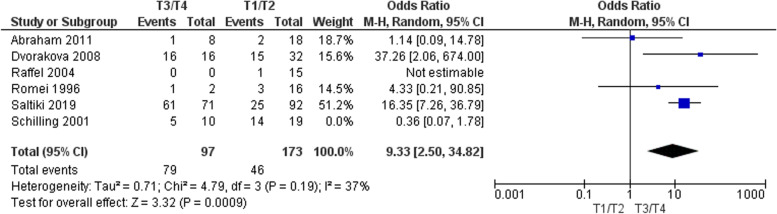
Pooled analysis of the impact of T-stage with respect to recurrence in sMTC

Six studies totalling 299 patients were considered for meta-analysis [4, 8, 12–14, 17]. The pooled analysis is shown in Fig. 2. Patients with T3/T4 disease were significantly more likely to have recurrent disease in comparison with those with T1/T2 disease; OR 9.33 (95% CI 2.5 – 34.82) *p* = 0.0009. There was evidence of significant statistical heterogeneity when all 6 studies were pooled (I^2^ = 81%; *P* = 0.0004). ‘One out’ sensitivity analysis showed that the study by Schilling et al*.* (13) was the main driver of this heterogeneity. This is possibly as a result of persistent rather than recurrent disease which was unaccounted for in the manuscript. This study was therefore subsequently removed. However, this did change the significance of the result. Further exclusion of the study by Abraham et al. (10), which included some patients with persistent disease (but we did not consider this as ‘recurrence’), completely removed statistical heterogeneity with associated increased effect size of higher T stage disease (data not shown). However, since exclusion of Schilling et al. alone met our pre-determined I^2^ threshold of < 50%, it was maintained in the analysis. The study by Raffel et al. did not include T3 or T4 disease and therefore no odds ratio was estimable.

#### N Stage

**Fig. 3:**
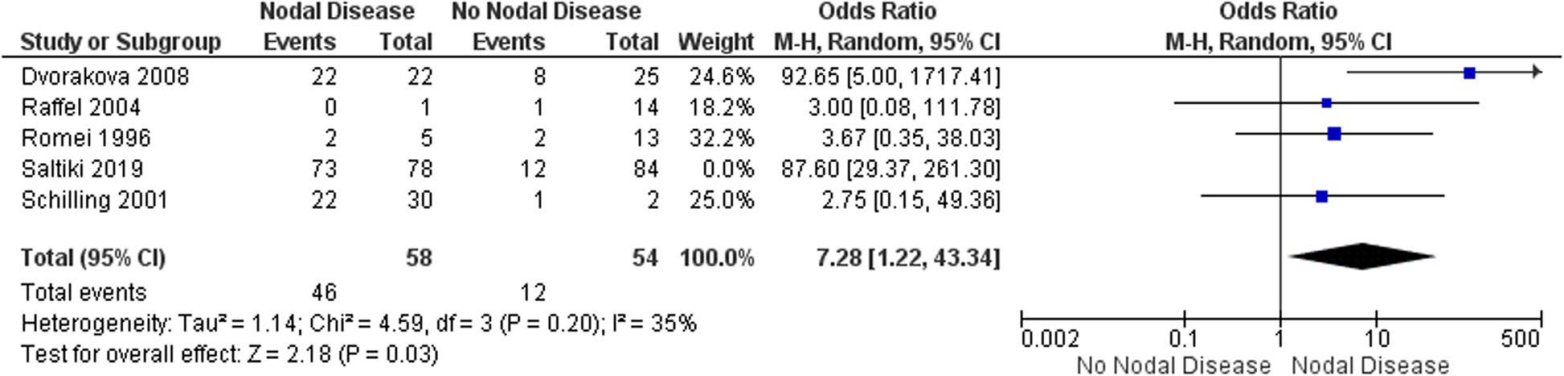
Pooled analysis of the impact of Nodal disease with respect to recurrence in sMTC

Five studies totalling 274 patients were considered for meta-analysis [4, 12–14, 17]. Patients with nodal disease at presentation were significantly more likely to develop recurrence in comparison to patients with node-negative disease at presentation; OR 7.28 (95% CI 7.2–43.3) *p* = 0.03 (Fig. 3). Again, there was evidence of significant statistical heterogeneity. This was driven by data from Saltiki et al. [4]. Removal of this study resulted in a reduction of the I^2^ from 65% *p* = 0.02 to 35% *p* = 0.20. The source of this statistical heterogeneity is unclear. Following discussion with the authors, it does not appear to be due to the presence of persistent disease. Its removal did not change the overall significance of our findings.

#### AJCC stage

**Fig. 4:**
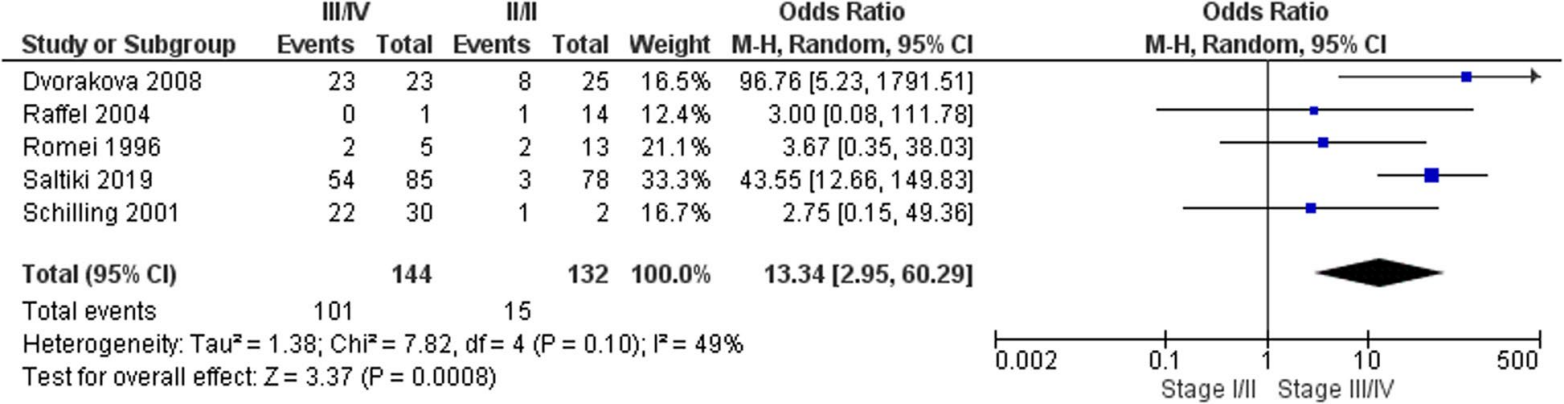
Pooled analysis of the impact of AJCC stage with respect to recurrence in sMTC

Five studies totalling 276 patients were included in this meta-analysis [4, 12–14, 17]. Patients with stage III/IV disease were significantly more likely to experience recurrence than those with stage I/II disease at presentation; OR 13.34 (95% CI 2.9 – 60.3) *p* = 0.0008 (Fig. 4).

#### Somatic RET mutation

**Fig. 5:**
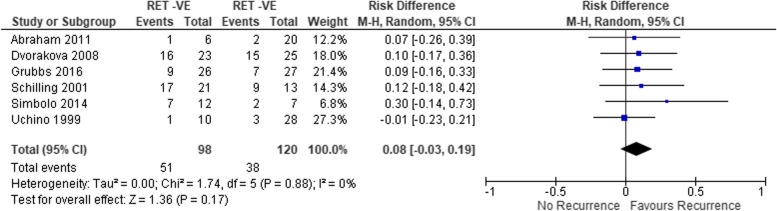
Pooled analysis of the impact of somatic RET mutation with respect to recurrence in sMTC

Data from six studies totalling 218 patients were included in the meta-analysis [8, 10–12, 14, 15]. The presence of a somatic RET mutation was not associated with recurrent disease in comparison to all other cases; OR 0.08 (95% CI -0.03–0.19) p = 0.17 (Fig. 5).

#### M918T mutation

**Fig. 6:**
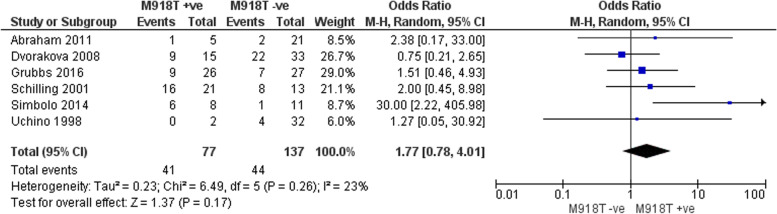
Pooled analysis of the impact of M918T with respect to recurrence in sMTC

Data from six studies totalling 213 patients were included in this meta-analysis [8, 10–12, 14, 16]. The presence of an M918T *RET* mutation was not associated with recurrent disease compared to all other cases; OR 1.77 (95% CI 0.804.0) *P* = 0.17 (Fig. 6).

#### RAS mutations, extrathyroidal extension, lymphovascular invasion, perineural invasion and extranodal extension

We were not able to extract any data on these characteristics for sMTC with respect to disease recurrence.

## Discussion

### Summary of results

This rigorous systematic review and meta-analysis has identified that in patients with sMTC who had an excellent response to their initial treatment, those with T3/4 disease, the presence of nodal disease and AJCC stage III/IV disease are at a higher risk of recurrence. We did not find evidence that either somatic *RET* mutations or the specific *RET* M918T were associated with recurrent disease.

This is broadly consistent with other literature, including another recently published systematic review by Vuong et al. [7]. However, there are some differences, particularly with respect to *RET* and *RAS* mutations. One key finding from this review is that without a standard definition of disease recurrence in sMTC, the datasets available on this topic are heterogeneous. Much of the literature fails to differentiate recurrent from persistent disease which are two very different subsets of groups with different management strategies. This significantly contaminates the literature on disease recurrence. Furthermore, as can be seen in Fig. 1, a large number of studies do not report recurrence with respect to clinico-pathological characteristics in a way that allows for the pooling of data and only one author contacted provided data in this manner.

### Clinicopathological characteristics

Whilst it is not surprising that patients with later stage disease are more likely to experience adverse outcomes in comparison to earlier stage disease, this is the first study to our knowledge to show that this is the case in sMTC in the context of recurrence. The majority of sMTCs present with locoregional and metastatic disease [2] and therefore many patients are already in higher risk categories for recurrence. Our findings are consistent with a large-scale analysis of the American Surveillance, Epidemiology and End Results (SEER) Program dataset. This has previously shown that survival correlates with local, regional and distant disease [18] and therefore it is reasonable to expect AJCC disease stage correlates with recurrent disease. What is still unanswered is which aspect of staging may be the most important factor. In our meta-analysis, stage III and IV AJCC staging had the highest odds ratio for recurrence rather than T3 or 4 disease or the presence of nodal disease alone. However, we cannot comment on degree to which each contributes to the risk of recurrence. 

### Mutational analyses

We did not demonstrate that patients with somatic *RET* mutations or the specific mutation *RET M918T* predicted a higher risk of recurrent disease. This contradicts the findings of a recent meta-analysis by Vuong et al*.* [7] which found that any *RET* mutation including M918T is associated with recurrent disease. There are a number of possible explanations for this discrepancy. Firstly, their definition of recurrence differed from our own. We specifically investigated recurrence as defined in our methods, whereas Vuong et al*.* included persistent disease in their analysis of recurrence. We elected not to class ‘persistent’ disease as recurrence, as the presence of persistent disease itself selects a group of patients who will already be managed differently once persistent disease is established. Secondly, the only two studies with clear positive results in their analysis were from Zedenius et al. [19] and from Elisei et al. [20]. Neither of these studies investigated recurrent disease but censored their outcomes if patients were ‘not cured’ or had ‘persistent disease’. This also affected other studies in their analysis by Cho et al. [21] and Moura et al*.* [22] and Cavedon et al. [23]. Finally, Chuang et al. [24] which had less than 10 patients was included in their study but was excluded in ours.

Two studies reported only *RET* M918T mutational status. These were included in the pooled analyses for both the overall *RET* and *RET* M918T mutation status [10, 14]. Their removal from the overall *RET* mutation analyses did not affect the significance of the result, although it did reduce I^2^ to 0%.

We attempted to pool data on *RAS* mutations. However, no study was eligible for inclusion as none reported on recurrent disease as we defined it. Of the three studies included in the Vuong et al*.* meta-analysis, two [25, 26] were contaminated with cases of persistent disease and therefore had a different case definition to the current study, and the other did not measure recurrent disease [23].

### Impact on initial surgical management and adjuvant therapy

The British Thyroid Association recommends that patients with sMTC should be offered a total thyroidectomy and central nodal clearance with lateral neck clearance if there is evidence of central nodal disease or ipsilateral lateral neck involvement [5]. Management of the contralateral neck is more controversial. The American Thyroid Association management guidelines suggest offering a contralateral neck dissection if basal calcitonin levels are > 200 pg/mL [1]. However, the British Thyroid Association do not make such an assertion noting that contralateral neck dissection may increase the chance of biochemical cure but have a limited impact on survival [5]. We have found that patients with T3/T4, N1 and stage III/IV disease are at increased risk of recurrence in those who have had an excellent response to treatment. Whilst decision-making in locally advanced disease can be straightforward with the need for radical resection of loco-regional disease, it remains to be seen if risk factors can be used to stratify patients into groups that may benefit from more aggressive surgical management.

### Limitations

Firstly, despite a large number of studies potentially eligible for inclusion on full text review, we were only able to extract data from 10 manuscripts. We found that whilst studies often included a number of outcome variables and may have reported recurrence, more often than not, they failed to report results in a way amenable to pooling for meta-analysis. For example, some studies provided unseparated data on cases of sporadic and familial MTC, whilst others did not report recurrence. Where recurrence was reported, it was often not reported in a way which allowed data to be pooled with respect to individual clinicopathological or mutational characteristics, or was confounded by persistent disease. We attempted to mitigate this by contacting authors for further data. We contacted 34 groups (totalling 39 manuscripts) offering the opportunity for inclusion of their work in this analysis. We received three replies with only one group able to provide data. This explains the absence of large datasets, including recently published studies [27] from groups who have contributed valuable work in this field. As a result, there may be some degree of publication bias given how few studies were included. However, this could not be formally assessed as there were inadequate numbers of included studies to accurately assess funnel plots or to perform regression-based assessments. Therefore, we are unable to comment on when patients recur, and patterns of recurrence. What is clear is that if a more in-depth risk profiling of recurrence in this rare disease is going to be established, it will require a multi-centre collaborative approach with open data sharing.

Secondly, there are large confidence intervals for the clinico-pathological characteristics we have explored. The exception for this is the mutational analyses where these intervals are smaller. Whilst we can be confident in the data presented, we have refrained from making firm assertions regarding the strength of these associations. We certainly cannot, from this data, explore the relationship between these characteristics and disease recurrence.

Thirdly, with recent advancements in imaging, more modern studies may be more likely to identify recurrence. Therefore, there may be an underestimation of recurrence in the earlier literature. However, only three of the average follow-up times recorded was less than five years and all were more than 2.5 years. Therefore, many patients with recurrent disease would have had time to develop identifiable disease to be included in this study.

Additionally, it was unclear how some cases were collected, such as whether they were consecutive. This could be particularly important, for example, in data looking retrospectively at *RET* mutations that rely on the availability of stored tissue. It may be that more tissue is available in larger tumour specimens that themselves may indicate more aggressive disease and therefore be subject to selection bias.

Finally, one potential criticism of this review is the omission of calcitonin from analyses. Large series suggest that the failure to achieve biochemical remission is an important predictor of recurrent disease in familial and non-familial disease [28]. However, throughout this review, we have been concerned regarding the definitions of recurrence throughout the literature. A failure to achieve biochemical remission within 6 months suggests persistent disease, as opposed to recurrence following biochemical cure. Therefore, this group of patients are a subgroup in their own right that merit investigation regarding factors contributing to disease recurrence. Chen et al.have recently suggested that the early ratio of pre and post-operative calcitonin can predict which patients develop structurally identifiable disease following a biochemically incomplete response to treatment and this requires validation in other cohorts [29]. With respect to allcomers with sMTC, we suggest that in addition to overall and disease-free survival, progression-free survival would be a particularly important outcome measure to consider in any future trials. This will at least partly account for stable persistent vs progressive persistent disease in patients with a biochemically incomplete response to treatment.

## Conclusions

Patients with T3/T4, node positive and AJCC stage III/IV sMTCs are at increased risk of recurrence following an excellent response to initial therapy compared with their early disease counterparts. There are a number of limitations in the sMTC literature with regards to outcome reporting in recurrent disease and future research should focus on collaboration to enable further comparisons with larger datasets with standardised definitions of recurrence. Whilst disease stage is a significant prognostic factor with respect to recurrence, in the future there may be subgroups who could be targeted for more radical upfront treatment, early systemic treatment or more intense surveillance.

## Supplementary Information

**Additional file 1.**

## Data Availability

The datasets used and/or analysed during the current study are available from the corresponding author on reasonable request.
