# Supplementary material for: Sporadic medullary thyroid cancer: a systematic review and meta-analysis of clinico-pathological and mutational characteristics predicting recurrence
Source: Thyroid Res. 2022 Jul 22;15:12. doi: 10.1186/s13044-022-00130-8 (PMC9306201; doi:10.1186/s13044-022-00130-8)
Supplement: Supplementary file 1 — Additional file 1. [file 13044_2022_130_MOESM1_ESM.docx]

**Appendix One:**

Database: Ovid MEDLINE(R) and In-Process & Other Non-Indexed Citations <1946 to May 09, 2020>

Search Strategy:

--------------------------------------------------------------------------------

1     exp Thyroid Neoplasms/

2     exp Carcinoma, Medullary/

3     1 and 2 (2610)

4     medullary thyroid.mp.

5     MTC.mp.

6     4 or 5

7     sporadic.mp.

8     sMTC.mp.

9     3 or 6

10     7 and 9

11     8 or 10

12     exp Disease-Free Survival/ or exp Progression-Free Survival/

13     exp Treatment Outcome/

14     exp Prognosis/

15     exp Recurrence/ or exp Neoplasm Recurrence, Local/

16     prognos*.mp.

17     relaps*.mp.

18     recurren*.mp.

19     12 or 13 or 14 or 15 or 16 or 17 or 18

20     11 and 19

21     limit 20 to english language

22     limit 21 to "review articles"

23     21 not 22

24     limit 23 to last 25 years

25     limit 24 to "reviews (best balance of sensitivity and specificity)"

26     24 not 25

27     limit 26 to (clinical conference or comment or editorial or letter or "review")

28     26 not 27

***************************
